# Bacterial Apoptosis-Like Death through Accumulation of Reactive Oxygen Species by Quercetin in *Escherichia coli*

**DOI:** 10.4014/jmb.2403.03057

**Published:** 2024-06-14

**Authors:** Min Seok Kwun, Dong Gun Lee

**Affiliations:** School of Life Sciences, BK 21 FOUR KNU Creative BioResearch Group, College of Natural Sciences, Kyungpook National University, Daegu 41566, Republic of Korea

**Keywords:** Apoptosis-like death, *Escherichia coli*, quercetin, reactive oxygen species

## Abstract

The antimicrobial activity of the natural compounds from plant and food have well discovered since the interest on the beneficial effect of the natural compounds was risen. Quercetin, a flavonoid derived from vegetables, including onions, red leaf lettuces and cherries has been studied for diverse biological characteristics as anti-cancer and anti-microbial activities. The aim of current study is to investigate the specific antibacterial modes of action of quercetin against *Escherichia coli*. Quercetin decreased the *E. coli* cell viability and induced the severe damages (oxidative stress, DNA fragmentation) leading to cell death. Reactive oxygen species (ROS) generation was observed during the process, which we confirmed that oxidative stress was the key action of antibacterial activity of quercetin exerting its influence potently. Based on the results of Annexin V and Caspace FITC-VAD-FMK assay, the oxidative damage in *E. coli* has led to the bacterial apoptosis-like death in *E. coli*. To sum up, the contribution of ROS generation exerts crucial impact in antibacterial activity of quercetin.

## Introduction

*Escherichia coli* is a rampant bacterium which exists in the intestines of humans and warm-blooded animals [1]. Typically, *E. coli* is a beneficial bacterium that assists in nutrient absorption and digestion. However, several strains can act fatal to human body inducing lethal diseases even leading to death. Pathogenic strains such as Enterohemorrhagic coli (EHEC), Enteropathogenic coli (EPEC), and Enterotoxigenic coli (ETEC) can be considered as a serious threat to the human body [2]. Among these, *E. coli* O157:H7, a strain of Enterohemorrhagic *E. coli*, has globally caused food and waterborne diseases [3]. Currently there are no specific treatments for *E. coli* O157 infections and its antibiotic resistance. While antibiotics like ampicillin and ciprofloxacin have been used against in the past, their use is now limited due to the increased risk of developing hemorrhagic colitis [4]. Given this situation, the discovery of antibiotics for the antimicrobial activity against *E. coli* O157 is necessary.

Flavonoids, known for their antioxidant activity, are mostly extracted from edible sources from plants including fruits, vegetables [5]. Recent studies have focused on the properties of flavonoids as well as their derivatives for biological purposes [6]. Meanwhile, the antimicrobial activity of flavonoids is highlighted, with their diverse biological activities. When apoptotic factors induction is regulated along with generation of reactive oxygen species (ROS), yeast apoptosis is known to be induced by flavonoids [7]. One of the most common flavonoids, quercetin (3,3',4',5,7-pentahydroxyflavone) is a natural product derived from 2-phenylchrome-4-one. This flavonol compound has a 3-hydroxyflavone backbone structure, included in a major subclass of flavonoids. Quercetin is a flavonol-based natural product with a 3, 5, 7, 3', 4'-pentahydroxy flavon structure [8]. Quercetin exhibits antibacterial activity against pathogenic microorganisms such as bacteria and fungi. Quercetin's antibacterial activity plays an important role in inhibiting growth by destroying bacterial cell walls and cell membranes [9]. Potent microbial activity of quercetin is well reported and further investigations against bacterial, fungal and viral pathogens are still conducted [10]. As such, while the antibacterial activity of quercetin is known, the intracellular mechanism of quercetin remains poorly understood, necessitating further research.

ROS is one of the highly toxic oxidants, classified as a type of free radical [11]. ROS consists of oxygen-based compounds, including hydroxyl radical (OH^-^), superoxide (O_2_^-^), and hydrogen peroxide (H_2_O_2_). H_2_O_2_ is normally generated by the autoxidation of redox enzymes and, if accumulated a lot, could cause damage on intracellular components and DNA. Therefore, organisms possess various scavenging enzymes like catalases and to protect themselves from H_2_O_2_. Moreover, O_2_^-^ is a precursor to active free radicals and is formed under the reaction of cellular oxidation. O_2_^-^ plays an important role in the formation of other ROS like singlet oxygen and trigger the critical damage of lipids, proteins, and DNA [12]. OH^-^ exerts potent oxidative damage in cell by reacting with DNA components and both purine, pyrimidine bases along with deoxyribose backbone are damaged as a result [13]. In addition, OH^-^ is considered as the most powerful ROS that induces the severe damage, leading to cell death. ROS is generated as part of metabolic activities within cells, and some ROS molecules are involved in cell signaling or cellular protection [14]. The generation and concentration of ROS within cells must be strictly regulated. Excessive ROS can induce oxidative damage in *E. coli*, leading to dysfunction in GSH, which protects bacteria from oxidative stress and ROS [15]. This can alter the membrane potential that protects bacteria from antimicrobial agents, ultimately disrupting the defense system and causing cell death. This study elucidated the antibacterial activity of quercetin against *E. coli*, with a particular focus on investigating the effects and mechanisms of ROS in *E. coli*.

## Materials and Methods

### Quercetin Preparation

Quercetin (Sigma-Aldrich, USA) was dissolved in dimethyl sulfoxide (DMSO). *E. coli* (ATCC25922) was obtained from the American Type Culture Collection (ATCC, USA). For all experiments, bacterial cells were grown in Luria–Bertani (LB, BD Pharmingen, USA) agar plates at 37°C and incubated in LB broth under aerobic conditions at 37°C and 120 ×*g*. Bacterial strains in the exponential phase were harvested and then resuspended in phosphate-buffered saline (PBS, pH 7.4).

### Detection of Intracellular ROS Accumulation and Glutathione Assay

2’,7’-dichlorodihydrofluorescein diacetate (H_2_DCFDA) (Molecular Probes) was used for indicating ROS generation. Bacterial cells were incubated after treatment of quercetin (10 μg/ml) at 37°C for 4 h. Then, the cell centrifugation at 12,000 ×*g* for 5 min was done and the cell pellets were resuspended in 1 μM H_2_DCFDA at 37°C for 1 h. After twice washing with PBS, cells were analyzed with a FACSVerse flow cytometer. In glutathione assay, the incubation with quercetin for 4 h at 37°C was done. To determine the total content of glutathione, the resuspension of cell pellets in 5% SSA solution was held and caused the precipitation of proteins and went through freeze-thaw cycles to lyse. The lysate incubation was held for 5 min at 4°C and the supernatant was used afterwards. 2-vinylpyridine was treated for 1 h at room temperature. The measurement in assay was according to the Bergmeyer description of glutathione reductase enzymatic recycling method. A microtiter ELISA Reader was applied for analyze [16].

### DNA Fragmentation, Caspase-Like Protein Activation Analysis

FITC-VAD-FMK (Promega, USA) was applied for determination of the caspase-like activity. *E. coli* cells were incubated with 10 μg/ml quercetin. the cell solutions were then twice washed and incubated with dye for 20 min. A FACSVerse flow cytometer was used for verification [17]. Moreover, to detect the DNA fragmentation by labeling the 3'- hydroxyl termini in the ds DNA breaks that were generated during apoptosis, the cells were first incubated at 37°C for 2 h, and then fixed with 2% paraformaldehyde for 1 h and incubated with a permeabilization solution, which contained 0.1% of sodium citrate and 0.1% Triton X-100, on ice for 2 min. Next, the cells were incubated with the TUNEL reaction mixture came with an *in situ* cell death detection kit (Sigma-Aldrich) at 37°C for 1 h. Fluorescence intensities were measured by FACSVerse [18].

### Analysis of Phosphatidylserine (PS) Exposure and Membrane Depolarization

PS exposure was detected using the Annexin V–FITC apoptosis detection kit (BD Pharmingen, USA). Cells were treated with quercetin (10 μg/ml) or norfloxacin (1.5 μg/ml) were incubated for 2 h at 37°C. After incubation, the cells were collected and resuspended in 100 μl of 1× binding buffer, followed by the addition of 50 μl/ml of Annexin V–FITC to the cell suspensions. The mixtures were then incubated at room temperature for 15 min in the dark. The cells were analyzed using a FACSVerse flow cytometer [19]. Moreover, the bis-(1,3-dibutylbarbituric acid) trimethine oxonol [DiBAC_4_(3)] (Molecular Probes, USA) was used to evaluate membrane depolarization. Cells were treated for 2 h at 37°C. Following this incubation, the cells were washed with PBS and stained with 5 μg/ml DiBAC_4_(3). Fluorescence intensity was analyzed using a FACSVerse flow cytometer [20].

### Statistical Analysis

Each experiment was performed in triplicate, and the data are presented as the means ± standard deviation. A comparison of group statistics was conducted using an analysis of variance (ANOVA) followed by Tukey’s test comparisons of three groups using SPSS software (version 25; SPSS/IBM). Intergroup differences were regarded statistically significant at *P* < 0.5 and *P* < 0.01 and *P* < 0.001.

## Results and Discussion

### ROS Generation after Quercetin Treatment

**Fig. 1:**
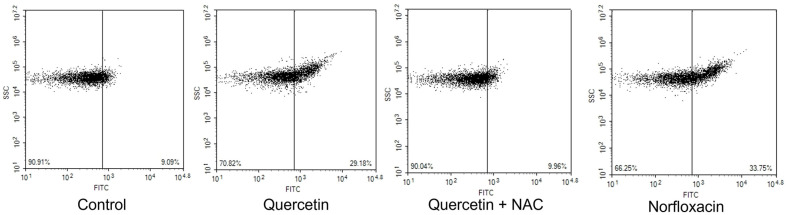
Flow cytometric analysis of ROS generation was performed with H_2_DCFDA staining in *E. coli*.

ROS, associated with O_2_^-^, H_2_O_2_, and OH^-^ has effects on diverse cellular interaction and needs regulation due to possible cause of damage in cells related to survival [21]. Aerobic organisms generate ROS and it is essential for cell functioning because it is a mediator of intracellular signaling cascades. But ROS accumulation could lead to excessive oxidative stress, occurring programmed cell death and necrosis. For verification of impact of ROS on the bacterial cell death process induced by quercetin, a ROS scavenger NAC, which suppresses cytotoxicity and apoptosis was used and proved that it did not harm the cell by itself. While the untreated samples displayed the relative fluorescence intensity of 9.09, the quercetin-treated sample’s intensity was clearly increased to 29.18 with positive control, norfloxacin displaying 33.75. For verification of NAC in bacterial cells, NAC was pretreated and ROS generation was successfully blocked, showing 9.96 (Fig. 1).

### Oxidative Damage Induced by Quercetin and Following DNA Fragmentation

**Fig. 2:**
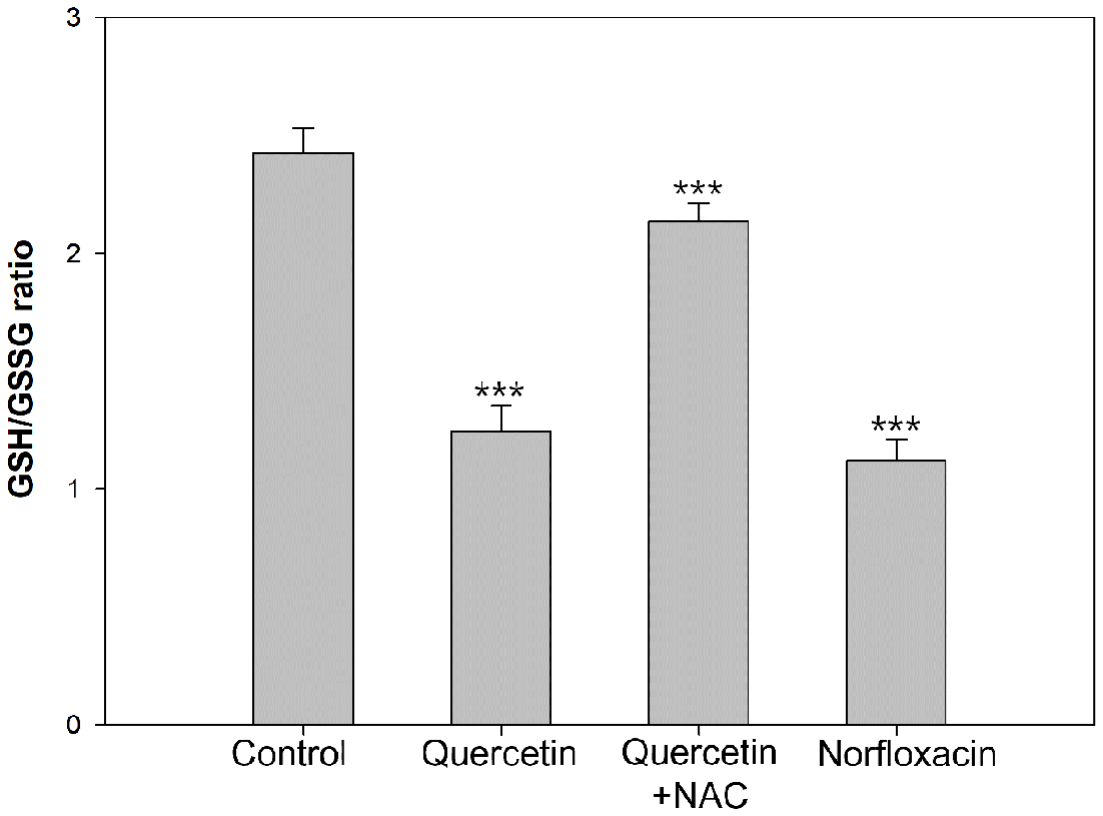
GSH/GSSG ratio was observed in *E. coli* measured with glutathione assay. Experiments were performed in triplicate and the results represent the average, standard deviation, and P values from three different analyses (*P < 0.5; **P < 0.01; ***P < 0.001).

**Fig. 3:**
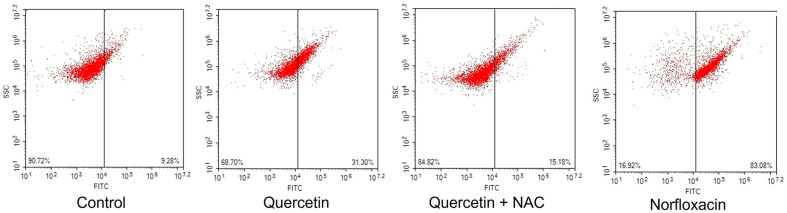
Analysis of DNA fragmentation was performed with terminal deoxynucleotidyl transferase dUTP nick end labeling (TUNEL) in *E. coli*.

Glutathione regulates the maintenance of redox homeostasis and function of cellular processes is controlled under its influence. The role of cellular ROS scavenger is held by glutathione and variation of its level induces ROS increment, which causes redox level decrease. In glutathione assay, increase in GSSG ratio to GSH confirms damage in cellular antioxidative system [22]. In this experiment, quercetin causes a redox level decrease in *E. coli*, after induction of ROS generation in the previous stage. Control cells which were untreated show a ratio of GSH/GSSG as 2.3, and quercetin-treated cells displayed a ratio of 0.9, and norfloxacin-treated cells were recorded as a ratio of 0.8. However, under pre-treatment of NAC, the ratio of quercetin-treated were increased to ratio of 1.4 (Fig. 2). ROS generation from quercetin treatment can disrupt the antioxidant system, causing decrease in redox levels. NAC contributes to blockage of antioxidant system by quercetin and this indicates the prevention can be held in the initial stage with protection of cellular homeostasis. Moreover, accumulated oxidative damage can exert lethal cellular disruption in DNA, possibly leading to serious damage like fragmentation [23]. For DNA damage measurement, TUNEL assay was confirmed, which binds to free 3’-OH ends of cleaved DNA. In Fig. 3, Cells without any treatment showed relative intensity of 9.28. When quercetin was treated, 31.30 was monitored and norfloxacin treatment was monitored as 83.08. When NAC was pre-treated, relative intensity decreased to 15.18. Following results indicate that ROS generation from quercetin can cause lethal damage to cells, exerting oxidative damage, disrupting cellular homeostasis, leading to DNA damage.

### Apoptosis-Like Death Hallmarks: Caspase-Like Protein Activation, Membrane Depolarization and Phosphatidylserine Exposure

**Fig. 4:**
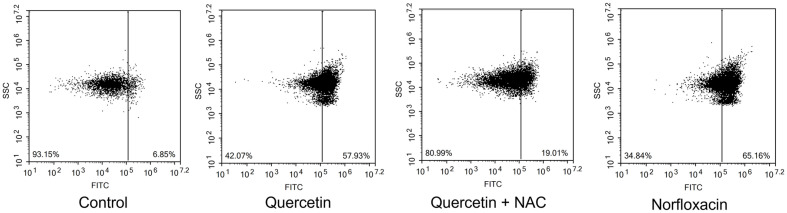
Flow cytometric analysis of caspase-like activity was measured by FITC-VAD-FMK in *E. coli*.

**Fig. 5:**
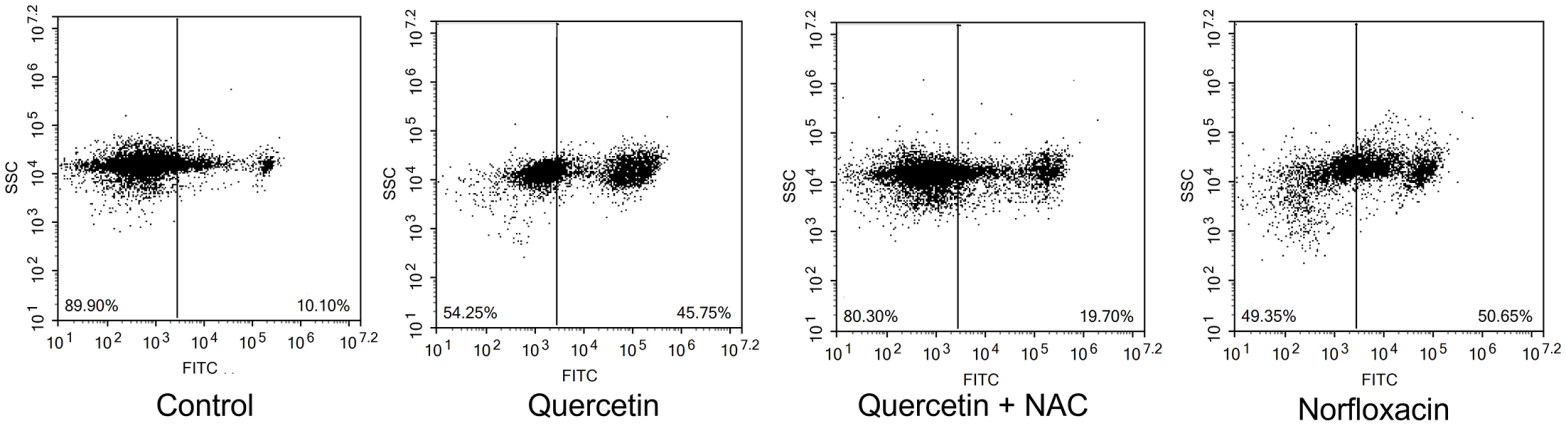
Flow cytometric analysis of membrane depolarization was measured by DiBAC_4_(3) in *E. coli*.

**Fig. 6:**
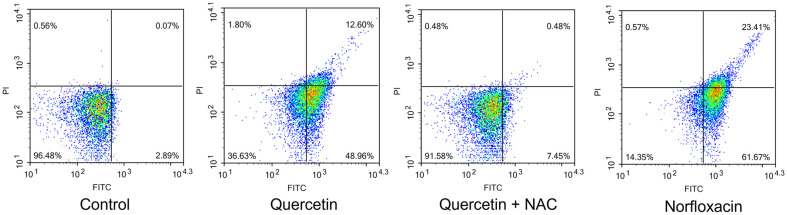
Flow cytometric analysis of PS exposure was performed with Annexin V/propidium iodide double staining in *E. coli*.

Lethal damage in cells leads to cell death, displaying various types of pathways. DNA damage cause cells to lethal state, triggering cell inactivation, and eventually apoptosis. CaspACE FITC VAD-FMK, a fluorescent pan caspase inhibitor, was applied for verification. When inhibitor is delivered inside the cells, this agent irreversibly binds to activated caspases, and the fluorescence are detected. This caspase-like protein activation enables detection of apoptosis-like death in bacterial cells [24]. In Fig. 4, cells without any treatment were recorded with the relative fluorescence of 6.85 in a positive population. When quercetin was treated, the intensity rose to 57.93, showing similar statistics to cells treated with norfloxacin, 65.16. When NAC was pre-treated to cells, the fluorescence decreased to 19.01. Moreover, when cells undergo apoptosis, specific hallmark are displayed including membrane depolarization, detecting from DiBAC_4_(3) dye. DiBAC_4_(3) enables entrance of depolarized cells binding to intracellular proteins or membrane and fluorescence increase is monitored with a red spectral shift. Therefore, anionic dye can additionally enter, followed by verification of fluorescence increase [25]. In Fig. 5, under comparison between no treatment, quercetin and norfloxacin-treated cells, notable variation in fluorescence intensity was detected. From initial intensity of untreated cells to that of 10.10, the fluorescence increased to 45.75 and 50.65. Under Cohex pretreatment, the intensity did not increase much, showing 19.70. Annexin V/PI double staining is a certain detection kit of apoptosis-like death occurrence. When Annexin V binds to externalized phosphatidylserine (PS), the intactness of cell membrane integrity is detected. Since intactness of plasma membrane indicates apoptosis, this differentiates the cell death type between apoptosis and necrosis, mainly by PI fluorescence. Under occurrence of apoptosis-like death, Annexin V is displayed as positive with PI negative. Necrosis is indicated with Annexin V and PI both positive. In quercetin- or norfloxacin-treated cells, Annexin V relative fluorescence is displayed 2.89 to 48.96 and to 61.67, respectively. The both positive quadrant, indicating occurrence of necrosis or late apoptosis varied significantly, from 0.07 to 12.60 and 23.41 for quercetin- and norfloxacin-treated cells, respectively. Meanwhile, Annexin V negative and PI positive quadrant indicating clear necrosis, was displayed as 0.07, 1.80 and 0.57. From this result, it can be speculated that necrosis occurrence did not take place in the experiment (Fig. 6). When NAC was previously treated, the statistics exhibited different numbers compared to quercetin and norfloxacin-treated cells, indicating the contribution of ROS to eventually apoptosis-like death, displaying the destructive damage of ROS intracellularly.

Current experiment was designed for investigation of bacterial mode of action of quercetin, which its antibacterial activity was previously confirmed. Under the generation of ROS from widely-derivated flavonoid in *E. coli*, the bacterial cells undergo destructive oxidative damage, leading to disruption of cellular homeostasis, including redox system. Under the glutathione depletion from initial ROS generation, DNA damage occurred, resulting in fragmentation in *E. coli*. Following the damage from intracellular components, the bacterial cell eventually led to apoptosis-like death and several hallmarks were observed including depolarization of membrane, activation of caspase-like protein and PS exposure. For verification of effect of ROS throughout the whole process, NAC, an intense ROS scavenger was applied for pre-treatment and indicated that eventual cell death results from initial generation of ROS from quercetin. To sum up, quercetin contributed to apoptosis-like death in *E. coli* through oxidative damage induced by accumulation of ROS.
